# Association of Female Genital Schistosomiasis With the Cervicovaginal Microbiota and Sexually Transmitted Infections in Zambian Women

**DOI:** 10.1093/ofid/ofab438

**Published:** 2021-08-22

**Authors:** Amy S Sturt, Emily L Webb, Lisa Himschoot, Comfort R Phiri, Joyce Mapani, Maina Mudenda, Eyrun F Kjetland, Tobias Mweene, Bruno Levecke, Govert J van Dam, Paul L A M Corstjens, Helen Ayles, Richard J Hayes, Lisette van Lieshout, Isaiah Hansingo, Suzanna C Francis, Piet Cools, Amaya L Bustinduy

**Affiliations:** 1 Department of Clinical Research, London School of Hygiene and Tropical Medicine, London, UK; 2 MRC International Statistics and Epidemiology Group, London School of Hygiene and Tropical Medicine, London, UK; 3 Department of Diagnostic Sciences, Faculty of Medicine and Health Sciences, Ghent University, Ghent, Belgium; 4 Zambart, Lusaka, Zambia; 5 Department of Obstetrics and Gynecology, Livingstone Central Hospital, Livingstone, Zambia; 6 Department of Infectious Diseases, Oslo University Hospital, Oslo, Norway; 7 University of KwaZulu-Natal, Durban, South Africa; 8 Department of Virology, Parasitology, and Immunology, Ghent University, Merelbeke, Belgium; 9 Department of Parasitology, Leiden University Medical Center, Leiden, the Netherlands; 10 Department of Cell and Chemical Biology, Leiden University Medical Center, Leiden, the Netherlands

**Keywords:** cervicovaginal microbiota, female genital schistosomiasis, *Schistosoma haematobium*, sexually transmitted infection

## Abstract

**Background:**

The cervicovaginal microbiota, including sexually transmitted infections (STIs), have not been well described in female genital schistosomiasis (FGS).

**Methods:**

Women (aged 18–31, sexually active, nonpregnant) were invited to participate at the final follow-up of the HPTN 071 (PopART) Population Cohort in January–August 2018. We measured key species of the cervicovaginal microbiota (*Lactobacillus crispatus*, *L. iners*, *Gardnerella vaginalis*, *Atopobium vaginae*, and *Candida*) and STIs (*Chlamydia trachomatis*, *Neisseria gonorrhoeae*, *Trichomonas vaginalis*, and *Mycoplasma genitalium*) using quantitative PCR (qPCR). We evaluated associations of the microbiota and STI presence and concentration with FGS (qPCR-detected *Schistosoma* DNA in any of 3 genital specimens).

**Results:**

The presence and concentration of key cervicovaginal species did not differ between participants with (n = 30) or without FGS (n = 158). A higher proportion of participants with FGS had *T. vaginalis* compared with FGS-negative women (*P* = .08), with further analysis showing that *T. vaginalis* was more prevalent among women with ≥2 *Schistosoma* qPCR-positive genital specimens (50.0%, 8/16) than among FGS-negative women (21.5%, 34/158; *P* = .01).

**Conclusions:**

We found weak evidence of an association between the presence of *T. vaginalis* and FGS, with a stronger association in women with a higher-burden FGS infection. Additional research is needed on potential between-parasite interactions, especially regarding HIV-1 vulnerability.

Female genital schistosomiasis (FGS) is caused when eggs from the waterborne parasite *S. haematobium* are entrapped in genital tissue [1]. FGS is prevalent in Sub-Saharan Africa and is associated with adverse reproductive outcomes, including ectopic pregnancy, infertility, and prevalent HIV-1 [1, 2]. The cervicovaginal environment has been described as “optimal” when it is dominated by lactic acid–producing lactobacilli, commensal microorganisms that adhere to an intact vaginal squamous epithelium [3, 4], protecting against pathogens by acidifying the vagina and producing antimicrobial substances such as bacteriocins [3]. Bacterial vaginosis (BV) and vulvovaginal candidiasis are examples of “nonoptimal” microbiota. BV is prevalent in women in Sub-Saharan Africa [5, 6] and is characterized by a shift from lactobacilli dominance to an increase in anaerobic species or yeast [4]. BV has important sexual and reproductive health consequences, including increased risk of pelvic inflammatory disease [5]. Additionally, BV has been associated with adverse pregnancy outcomes such as preterm delivery [5, 7], a leading cause of under-5 mortality in Sub-Saharan Africa [8]. BV is also strongly associated with HIV-1 acquisition and other sexually transmitted pathogens infecting the genital tract [5, 9, 10]. Studies employing 16S rRNA sequencing to evaluate the cervicovaginal microbiota suggest that high-intensity urinary *S. haematobium* infection, in the absence of investigation for genital involvement, may alter cervicovaginal microbiota diversity [11]. However, the relationship between cervicovaginal microbiota and FGS is not well characterized.

Prevalent sexually transmitted infections (STIs) have been reported in women with [12] or in populations endemic for [13, 14] urinary schistosomiasis. However, evaluation of genital involvement (FGS) in studies of urinary *S. haematobium* and STI co-infection is not universally performed or reported [12–14]. The biopsy prevalence of FGS in participants with urinary *S. haematobium* infection ranges from 23.0% to 75.0% ([15, 16]), making the assumption of genital involvement (FGS) in urinary *S. haematobium* infection tenuous. A reference standard diagnostic does not exist for FGS. Thus, an FGS evaluation is often based on a combination of diagnostic tests including circulating anodic antigen (CAA), urine microscopy, colposcopy, tissue-based diagnostics (biopsy, Papanicolaou smear, wet prep), and polymerase chain reaction (PCR) [17–19]. Where an FGS evaluation has been performed, studies reporting PCR-defined FGS have either not investigated or reported STI prevalence [20–22] or STI prevalence has been correlated with visual FGS findings [23]. In this cross-sectional study, we utilized PCR to detect *Schistosoma* DNA in the female genital tract and evaluated the association of PCR-defined FGS with the concentration and presence of key markers of the cervicovaginal microbiota, including STI.

## METHODS

### Study Setting and Participants

The cross-sectional bilharzia and HIV (BILHIV) study [18] was nested in HPTN 071 (PopART), a cluster randomized trial to measure the impact of an HIV-1 combination prevention package [24]. In HPTN 071 (PopART), HIV-1 incidence was measured in a population cohort at baseline and 12, 24, and 36 months [24]. Between January and August 2018, after the 36-month HPTN 071 (PopART) visit, community workers made home visits to women expressing interest in the BILHIV study [18]. Eligible women were aged 18–31 years, not pregnant, sexually active, and resident in 1 of 2 urban communities that participated in HPTN 071 (PopART) in Livingstone, Zambia.

### Home and Clinic-Based Sample Collection

The home visit included written informed consent, a questionnaire, genital self-sampling (cervical and vaginal), and urine specimen collection, as previously described [18]. Enrolled women not currently menstruating were invited to attend Livingstone Central Hospital cervical cancer clinic, where midwives performed cervicovaginal lavage (CVL). After speculum insertion, a bulb syringe was used to flush normal saline (10 mL) across the cervix and vaginal walls for 1 minute. CVL fluid was collected from the posterior fornices (Supplementary Data). CVL and vaginal and cervical swab specimens were used for quantitative PCR (qPCR) detection of *Schistosoma*; cervical swabs were used for characterization of the microbiota and STI by qPCR; urine was used for detection of CAA and *S. haematobium* eggs by microscopy.

Cervicovaginal images were captured with a portable colposcope (MobileODT, Tel Aviv, Israel) and were evaluated (by E.F.K.) for any of the 4 recognized FGS cervicovaginal manifestations: grainy sandy patches, homogenous yellow sandy patches, rubbery papules, and abnormal blood vessels [25]. Women with at least 1 of these manifestations [25] or with any positive urine or genital *Schistosoma* diagnostic were treated free of charge with 40 mg/kg of praziquantel. Testing for STI was not performed at the point of care, and participants with suspected STI were offered syndromic management, as per local guidelines [26].

### HIV-1

Laboratory-based fourth-generation HIV-1 testing (Abbott Architect HIV Ag/Ab Combo Assay) was performed for HPTN 071 (PopART) Population Cohort participants at each study visit [24].

### Urine Microscopy and Circulating Anodic Antigen

Urine was centrifuged and examined by microscopy for *S. haematobium* eggs. The participant was considered positive if a pellet contained at least 1 *S. haematobium* egg [18]. A lateral flow assay utilizing up-converting reporter particles for the quantification of CAA was performed on urine samples, as previously described [18, 27]. Analyzing the equivalent of 417 µL of urine (wet reagent, UCAA*hT*417), a test result indicating a CAA value >0.6 pg/mL was considered positive [28].

### qPCR for Detection of Schistosoma DNA

Detection of the *Schistosoma*-specific internal-transcribed-spacer-2 (ITS2) target by qPCR was performed at LUMC, as previously described (Supplementary Data) [18, 29]. DNA extraction of 200 µL of CVL or cervical or vaginal swab fluid was done with QIAamp spin columns (QIAGEN Benelux, Venlo, the Netherlands) according to manufacturer’s guidelines. The qPCR output was reported in cycle threshold values (Ct values), and parasite DNA loads were categorized by the following prespecified values: high (Ct < 30), moderate (30 ≤ Ct < 35), low (35 ≤ Ct < 50), and negative (no amplification) [30].

### Cervicovaginal Microbiota Characterization and STI Detection

We quantified *Lactobacillus crispatus* as a key marker of vaginal health. Additionally, we characterized markers of a “nonoptimal” cervicovaginal microbiota (*Gardnerella vaginalis* and *Atopobium vaginae*), as well as *L. iners* (an enigmatic and highly prevalent lactobacillus), *Candida spp.* and STI (*Chlamydia trachomatis*, *Neisseria gonorrhoeae*, *Mycoplasma genitalium*, and *Trichomonas vaginalis).* STI were quantified by qPCR using the S-DiaCTNG (for *C. trachomatis* and *N. gonorrhea*) and S-DiaMGTV kits (for *M. genitalium* and *T. vaginalis*; Diagenode Diagnostics, Seraing, Belgium) on DNA from cervical swabs at Ghent University (Ghent, Belgium) according to the manufacturer’s instructions. Quantification of *A. vaginae*, *G. vaginalis*, *L. crispatus*, *L. iners*, and *Candida* species was performed in the Laboratory Bacteriology Research, Ghent, using LightCycler480, version 1.5 (Roche, Basel, Switzerland) (Supplementary Data). The concentration of each species was expressed as genome-equivalents per mL (ge/mL) [31].

### FGS Definitions

In this study, we employed various diagnostic tests to evaluate urinary schistosome infection (CAA and urine microscopy) and FGS (portable colposcopy and *Schistosoma* DNA on CVL and genital swabs). As previously described [32], participants were grouped by diagnostic test results into 3 mutually exclusive categories: FGS, at least 1 positive *Schistosoma* qPCR on a genital specimen (cervical swab, vaginal swab, and/or CVL); FGS negative, negative results on all diagnostic methods; probable FGS, genital *Schistosoma* qPCRs negative but urinary schistosomiasis positive (as defined above), in combination with 1 of 4 clinical findings suggestive of FGS on any colposcope-obtained photograph [25].

### Patient Consent

The study was approved by the University of Zambia Biomedical Research Ethics Committee (011-08-17), the Zambia National Health Research Authority, and the London School of Hygiene and Tropical Medicine Ethics Committee (14506). Permission to conduct the study was given by Livingstone District Health Office and the Livingstone Central Hospital superintendent. Participants provided written informed consent.

### Statistical Methods

All participants with FGS (n = 30) and all participants with probable FGS (n = 25) were selected for characterization of the cervicovaginal microbiota and STI by qPCR on cervical swabs. Three FGS-negative participants were selected for every FGS and probable FGS participant using a random number generator. The FGS-negative participants were frequency-matched by age to participants with FGS (age groups 18–19, 20–21, 22–23, 24–25, 26–27, 28–29, 30–31).

Participant characteristics were summarized by median and interquartile range (IQR) for continuous variables and by frequency and percentage for categorical variables. Differences in characteristics between FGS categories were evaluated using the Fisher exact or chi-square test. For cervicovaginal microbiota and STI species with at least 20% of sample results detectable by qPCR (ie, ≥20% prevalence), *P* values for comparison of presence, median (IQR), and log_10_ concentration mean between FGS and FGS-negative groups were calculated using the chi-square test, rank-sum test, and *t* test, respectively. For species with <20% prevalence, species presence was compared between FGS groups using the Fisher exact test. To enable investigation of potential confounding, concentrations of cervicovaginal microbiota with ≥20% prevalence were log-transformed to normalize their distribution, and linear regression was used to evaluate the association between FGS and mean log-concentration of each organism (ge/mL) in univariable and multivariable analysis. We developed a causal framework (Supplementary Figure 1) to inform our minimal adjustment set and adjusted for age, education, and community of residence. For all species, logistic regression was used to calculate the crude and adjusted odds ratio (OR) for presence vs absence by FGS group; due to the relatively low number of participants with detectable concentrations, logistic regression analyses only adjusted for age. Given the exploratory nature of this work, we did not correct for multiple comparisons.

Our primary analysis focused on the detection of *Schistosoma* DNA in the genital tract (FGS vs FGS negative). A secondary analysis compared the FGS and probable FGS groups with the FGS-negative participants. To evaluate the possible association between FGS burden and changes in presence and concentration of cervicovaginal key species, 2 ad hoc analyses were performed: (1) participants with ≥2 genital samples with detectable *Schistosoma* DNA were compared with the FGS-negative group, (2) participants with a moderate/high genital *Schistosoma* DNA concentration (Ct < 35 in at least 1 of 3 samples) were compared with the FGS-negative group. Data were analyzed using STATA 15.1 (Stata Corporation, College Station, TX, USA).

## RESULTS

A total of 603 eligible women were enrolled in the BILHIV study, and 213 (35.3%) were included in the present study (Supplementary Figure 1). Of those included, 14.1% (30/213) had FGS, defined by a positive genital *Schistosoma* qPCR from any of the following sites: 9.4% (20/213) cervical swab, 7.0% (15/213) vaginal swab, and 6.6% (14/211) CVL. In participants with FGS, portable colposcopy revealed sandy patches in 20% (6/30), abnormal blood vessels in 20% (6/30), and no clinical signs of FGS in 50% (15/30). Two women with FGS did not present to the clinic for portable colposcopy, and 1 had uninterpretable images. Of participants with FGS, 53.3% (16/30) had *Schistosoma* qPCR detected from multiple sites, and 53.3% (16/30) had moderate/high genital *Schistosoma* DNA loads; these groups overlapped by 12 participants. Twenty-five women had probable FGS, and 74.2% (158/262) of the women who were negative on all diagnostic tests were randomly selected for inclusion in this study. Consistent with the FGS definitions, portable colposcopy in 100.0% (25/25) of the participants with probable FGS showed FGS-associated cervicovaginal manifestations. In the majority of participants with probable FGS, portable colposcopy showed sandy patches (76.0%, 19/25), with 24% (6/25) of participants having abnormal blood vessels.

### Baseline Characteristics

The majority of the participants were married/cohabitating, had received some secondary education, and were using hormonal contraception (Table 1). At the conclusion of HPTN 071 (PopART), HIV-1 prevalence was 17.4% (37/213) among the women included in this study, and one-third had at least 1 STI (Table 1). Urinary schistosome infection, defined as either a positive urine microscopy (11.7%, 25/213) or positive CAA (20.7%, 44/213), was reported in 21.6% (46/213) of participants. There were differences between the 3 categories of FGS status for age (*P* = .002), marital status (*P* = .04), education (*P* = .06), and employment (*P* = .04), with participants in the probable FGS group being more likely to be older, employed, and married than FGS and FGS-negative participants. There was strong evidence of a difference in community of residence by FGS status (*P* < .001), with participants with FGS and probable FGS more likely to live in Community A than participants in the FGS-negative group (Table 1). Other characteristics were similar by FGS status.

**Table 1.** Baseline Characteristics of the Study Population (n = 213) by FGS Status

Participant Characteristics		FGS Negative, No. (%) (n = 158)	Probable FGS, No. (%) (n = 25)	FGS, No. (%) (n = 30)	P Value^a^
Age, median (IQR), y		23 (22–24)	27 (23–31)	22 (21–24)	.002^b^
Marital status	Single	72 (45.6)	4 (16.0)	13 (43.3)	.04
	Married or cohabitating	81 (51.3)	20 (80.0)	17 (56.7)	
	Divorced or separated	5 (3.2)	1 (4.0)	0 (0.0)	
Education (highest level)	None or any primary school	35 (22.2)	12 (48.0)	10 (33.3)	.06
	Any secondary school	1111 (70.3)	13 (52.0)	19 (63.3)	
	Trade, degree or higher	12 (7.6)	0 (0.0)	1 (3.3)	
District	Community A	66 (41.8)	20 (80.0)	22 (73.3)	<.01
	Community B	92 (58.2)	5 (20.0)	8 (26.7)	
Household members	1–3	51 (32.3)	4 (16.0)	12 (40.0)	.3
	4–5	61 (38.6)	13 (52.0)	8 (26.7)	
	6+	46 (29.1)	8 (32.0)	10 (33.3)	
Employment status	Not working	117 (74.1)	14 (56.0)	26 (86.7)	.04
	Working	41 (25.9)	11 (44.0)	4 (13.3)	
Sexual behavior characteristics					
Age at sexual debut, y	8–16	64 (40.5)	11 (44.0)	17 (56.7)	.4
	17–19	74 (46.8)	13 (52.0)	10 (33.3)	
	20–24	20 (12.7)	1 (4.0)	3 (10.0)	
Lifetime sexual partners	1	57 (36.1)	8 (32.0)	5 (16.7)	.3
	2	36 (22.8)	6 (24.0)	9 (30.0)	
	3	23 (14.6)	6 (24.0)	8 (26.7)	
	4+	42 (26.6)	5 (20.0)	8 (26.7)	
Currently sexually active^c^^,^^d^	No	26 (16.6)	2 (8.0)	3 (10.0)	.5
	Yes	131 (83.4)	23 (92.0)	27 (90.0)	
Condom use with last sex^e^	No	120 (76.9)	16 (66.7)	22 (73.3)	.6^f^
	Yes	36 (23.1)	8 (33.3)	8 (26.7)	
HIV-1 status	Not detected	132 (83.5)	20 (80.0)	24 (80.0)	.8
	Detected	26 (16.5)	5 (20.0)	6 (20.0)	
Any STI^g^	Not detected	106 (67.1)	14 (56.0)	18 (60.0)	.5
	Detected	52 (32.9)	11 (44.0)	12 (40.0)	
Contraceptive use					
Condoms	No	132 (83.5)	20 (80.0)	26 (86.7)	.8
	Yes	26 (16.5)	5 (20.0)	4 (13.3)	
OCP	No	148 (93.7)	22 (88.0)	29 (96.7)	.5
	Yes	10 (6.3)	3 (12.0)	1 (3.3)	
Injectable	No	82 (51.9)	13 (52.0)	15 (50.0)	1.0^f^
	Yes	76 (48.1)	12 (48.0)	15 (50.0)	
Implant	No	144 (91.1)	23 (92.0)	28 (93.3)	1.0
	Yes	14 (8.9)	2 (8.0)	2 (6.7)	
Any hormonal contraception^h^	No	58 (36.7)	8 (32.0)	10 (35.7)	.9^f^
	Yes	100 (63.3)	17 (68.0)	18 (64.3)	
Schistosomiasis-related factors					
Urine microscopy	Not detected	158 (100.0)	19 (76.0)	11 (36.7)	<.001^i^
	Detected	0 (0.0)	6 (24.0)	19 (63.3)	
Urine CAA	Negative	158 (100.0)	0 (0.0)	11 (36.7)	<.001^i^
	Positive	0 (0.0)	25 (100.0)	19 (63.3)	

Abbreviations: CAA, circulating anodic antigen; FGS, female genital schistosomiasis; IQR, interquartile range; OCP, oral contraceptive pill; STI, sexually transmitted infection.

^a^Fisher’s exact *P* value unless otherwise indicated.

^b^Kruskal-Wallis *P* value.

^c^Any sexual activity in the last 6 months.

^d^Participants who responded with “no answer” (n = 1) are not shown in the table.

^e^Participants who responded with “no answer” (n = 3) are not shown in the table.

^f^Chi-square *P* value.

^g^Any STI defined as the presence of at least 1 of *N. gonorrhoeae*, *C. trachomatis*, *M. genitalium*, or *T. vaginalis.*

^h^Any hormonal contraception is defined as use of injectable agents, implants, or oral contraceptive pills.

^i^Part of the definition for FGS categories.

### Primary Comparison: FGS vs FGS Negative

**Figure 1.**
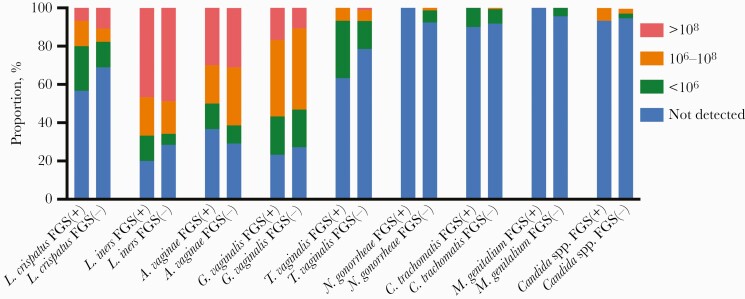
Stacked bar chart of concentrations of cervicovaginal microbiota and taxa causing sexually transmitted infection by female genital schistosomiasis status. Abbreviation: FGS, female genital schistosomiasis.

Concentrations of evaluated species are shown in Figure 1. Compared with FGS-negative women, there was no evidence of a difference in the presence or concentration of cervicovaginal *L. crispatus*, *L. iners*, *A. vaginae*, *G. vaginalis*, or *C. albicans* in participants with FGS (Table 2). A higher proportion of participants with FGS had *T. vaginalis* present, although there was not strong evidence of an association (OR, 2.11; 95% CI, 0.92–4.86; *P* = .08) (Supplementary Table 1). This result was similar after adjusting for age (Supplementary Table 1). Otherwise, compared with FGS-negative women, the presence and concentration of other STIs were similar in women with FGS (Table 2).

**Table 2.** Presence and Concentration of Vaginal *Lactobacilli*, Other Key Microbiota, and Sexually Transmitted Infection, Overall and by FGS Status

Organism	No. (%) and Concentration^a^	All Participants (n = 213)	FGS Negative (n = 158)	FGS (n = 30)	P Value^a^
*L. crispatus*	Presence	69 (32.4)	49 (31.0)	13 (43.3)	.19
	Median (IQR)	8.7×10^6^ (2.6×10^5^–4.2×10^8^)	1.1×10^7^ (2.6×10^5^–7.0×10^8^)	7.5×10^5^ (1.9×10^5^–2.7×10^7^)	.20
	Log-concentration mean	16.01	16.38	14.82	.19
*L. iners*	Presence	156 (73.2)	113 (71.5)	24 (80.0)	.34
	Median (IQR)	2.7×10^8^ (3.8×10^7^–1.5×10^9^)	2.8×10^8^ (3.9×10^7^–1.6×10^9^)	1.7×10^8^ (1.6×10^7^–1.0×10^9^)	.39
	Log-concentration mean	18.93	18.98	18.36	.33
*G. vaginalis*	Presence	156 (73.2)	115 (72.8)	23 (76.7)	.66
	Median (IQR)	7.7×10^6^ (8.3×10^5^–5.2×10^7^)	8.1×10^6^ (7.7×10^5^–4.8×10^7^)	3.8×10^6^ (6.8×10^5^–6.4×10^7^)	.93
	Log-concentration mean	15.85	15.74	15.91	.79
*A. vaginae*	Presence	152 (71.4)	112 (70.9)	19 (63.3)	.41
	Median (IQR)	5.8×10^7^ (8.2×10^6^–2.1×10^8^)	5.8×10^7^ (8.7×10^6^–2.0×10^8^)	3.0×10^7^ (1.06×10^6^–2.8×10^8^)	.83
	Log-concentration mean	17.31	17.33	17.06	.66
*T. vaginalis*	Presence	53 (24.9)	34 (21.5)	11 (36.7)	.08
	Median (IQR)	4.2×10^4^ (173.0–2.3×10^6^)	1.7×10^5^ (56.9–6.3×10^6^)	1.0×10^4^ (5030.0–4.5×10^5^)	.53
	Log-concentration mean	10.39	10.88	10.19	.69
Vaginal microbiota with prevalence <20%					
*N. gonorrheae*	Presence	13 (6.1)	12 (7.6)	0.0 (0)	.22
*C. trachomatis*	Presence	17 (8.0)	13 (8.2)	3 (10.0)	.72
*M. genitalium*	Presence	8 (3.8)	7 (4.4)	0 (0.0)	.60
*C. albicans*	Presence	12 (5.63)	8 (5.1)	2 (6.7)	.66

Abbreviations: FGS, female genital schistosomiasis; IQR, interquartile range.

^a^Concentrations are expressed in genome equivalents/mL.

^b^For species with >20% prevalence, *P* values for presence, median (IQR), and log-concentration mean are from the chi-square test, rank-sum test, and *t* test, respectively. For species with <20% prevalence, *P* values for presence are from the Fisher exact test.

Combining the FGS and probable FGS groups, participants with FGS/probable FGS similarly had a higher prevalence of *T. vaginalis* compared with FGS-negative participants (*P* = .05) (Supplementary Table 2). Otherwise, compared with FGS-negative women, the presence and concentrations of cervicovaginal microbiota and the presence of STI were similar in women with FGS/probable FGS compared with FGS-negative participants (Supplementary Table 2).

### Ad Hoc Analysis—Schistosoma DNA Concentration

In participants (n = 16) with FGS and a moderate/high genital *Schistosoma* DNA concentration (*Schistosoma* qPCR Ct < 35), the presence of *T. vaginalis* was higher than among the FGS-negative participants (*P* = .01) (Table 3). Women with FGS and a moderate/high *Schistosoma* DNA concentration (Ct < 35) had a higher mean concentration of *G. vaginalis* compared with FGS-negative women (*P* = .03) (Table 3).

**Table 3.** Ad Hoc Analysis of the Presence and Concentration of Vaginal *Lactobacilli*, Other Cervicovaginal Microbiota, and Sexually Transmitted Infection in Participants With a Moderate to High Concentration of *Schistosoma* DNA (Ct < 35) Compared With FGS-Negative Participants

Organism	No. (%) and Concentration^a^	FGS Negative (n = 158)	FGS With Ct < 35 (n = 16)	P Value^b^
*L. crispatus*	Presence	49 (31.0)	6 (37.5)	.60
	Median (IQR)	1.1×10^7^ (2.6×10^5^–7.0×10^8^)	1.2×10^6^ (3.0×10^5^–2.0×10^7^)	.28
	Log-concentration mean	16.38	14.27	.19
*L. iners*	Presence	113 (71.5)	11 (68.75)	.82
	Median (IQR)	2.8×10^8^ (3.9×10^7^–1.6×10^9^)	7.5×10^7^ (3.6×10^6^–7.5×10^8^)	.30
	Log-concentration mean	18.98	17.86	.21
*G. vaginalis*	Presence	115 (72.8)	13 (81.3)	.46
	Median (IQR)	8.1×10^6^ (7.7×10^5^–4.8×10^7^)	1.5×10^7^ (3.8×10^6^–3.1×10^8^)	.11
	Log-concentration mean	15.74	17.45	.03
*A. vaginae*	Presence	112 (70.9)	13 (81.3)	.38
	Median (IQR)	5.8×10^7^ (8.7×10^6^–2.0×10^8^)	1.6×10^8^ (1.6×10^7^–2.8×10^8^)	.70
	Log-concentration mean	17.33	17.38	.95
*T. vaginalis*	Presence	34 (21.5)	8 (50.0)	.01
	Median (IQR)	1.7×10^5^ (56.9–6.3×10^6^)	8085.0 (5210.0–1.0×10^6^)	.56
	Log-concentration mean	10.88	10.09	.70
Vaginal microbiota with prevalence <20%				
*N. gonorrheae*	Presence	12 (7.6)	0 (0.0)	.61
*C. trachomatis*	Presence	13 (8.2)	2 (12.5)	.63
*M. genitalium*	Presence	7 (4.4)	0 (0.0)	1.0
*C. albicans*	Presence	8 (5.1)	2 (12.5)	.23

Abbreviations: Ct, cycle threshold; FGS, female genital schistosomiasis; IQR, interquartile range.

^a^Concentrations are expressed in genome equivalents/mL.

^b^For species with >20% prevalence, *P* values for presence, median (IQR), and log-concentration mean are from the chi-square test, rank-sum test, and *t* test, respectively. For species with <20% prevalence, *P* values for presence are from the Fisher exact test.

### Ad Hoc Analysis—Clinical Disease Burden

In an ad hoc analysis, participants (n = 16) with a higher FGS burden, defined as ≥2 *Schistosoma* qPCR–positive genital specimens, had higher prevalence of *T. vaginalis* compared with FGS-negative participants (*P* = .01) (Table 4). There was also evidence of a difference in the median concentration and the mean log-concentration of *L. iners* (both *P* = .03) compared with FGS-negative women, with lower levels among the higher–FGS burden group (Table 4).

**Table 4.** Ad Hoc Analysis of the Presence and Concentration of Vaginal *Lactobacilli*, Cervicovaginal Microbiota, and Sexually Transmitted Infection in Participants With *Schistosoma* DNA Detected in ≥2 Genital Specimens Compared With FGS-Negative Participants

Organism	No. (%) and Concentration^a^	FGS Negative (n = 158)	≥2 PCR FGS (n = 16)	P Value^b^
*L. crispatus*	Presence	49 (31.0)	7 (43.8)	.30
	Median (IQR)	1.1×10^7^ (2.6×10^5^–7.0×10^8^)	1.9×10^6^ (7.7×10^4^–2.7×10^7^)	.32
	Log-concentration mean	16.38	14.90	.34
*L. iners*	Presence	113 (71.5)	10 (62.5)	.45
	Median (IQR)	2.8×10^8^ (3.9×10^7^–1.6×10^9^)	5.1×10^7^ (9.0×10^5^–1.1×10^8^)	.03
	Log-concentration mean	18.98	17.00	.03
*G. vaginalis*	Presence	115 (72.8)	14 (87.5)	.20
	Median (IQR)	8.1×10^6^ (7.7×10^5^–4.8×10^7^)	1.6×10^7^ (1.1×10^6^–3.6×10^8^)	.29
	Log-concentration mean	15.74	16.85	.16
*A. vaginae*	Presence	112 (70.9)	11 (68.8)	.86
	Median (IQR)	5.8×10^7^ (8.7×10^6^–2.0×10^8^)	1.6×10^8^ (2.7×10^7^–3.2×10^8^)	.33
	Log-concentration mean	17.33	18.04	.36
*T. vaginalis*	Presence	34 (21.5)	8 (50.0)	.01
	Median (IQR)	1.7×10^5^ (56.9–6.3×10^6^)	5680.0 (2967.5–1.0×10^6^)	.50
	Log-concentration mean	10.88	9.71	.56
Vaginal microbiota with prevalence <20%				
*N. gonorrheae*	Presence	12 (7.6)	0 (0.0)	.61
*C. trachomatis*	Presence	13 (8.2)	2 (12.5)	.63
*M. genitalium*	Presence	7 (4.4)	0 (0.0)	1.0
*C. albicans*	Presence	8 (5.1)	1 (6.3)	.59

Abbreviations: FGS, female genital schistosomiasis; IQR, interquartile range; PCR, polymerase chain reaction.

^a^Concentrations are expressed in genome equivalents/mL.

^b^For species with >20% prevalence, *P* values for presence, median (IQR), and log-concentration mean are from the chi-square test, rank-sum test and *t* test, respectively. For species with <20% prevalence, *P* values for presence are from the Fisher exact test.

## DISCUSSION

In this study, we describe the association of FGS with the cervicovaginal microbiota, including lactobacilli, *Candida* spp., markers of a “nonoptimal” cervicovaginal environment, and STI. We did not find evidence that the presence or concentration of key cervicovaginal species was associated with FGS. While FGS was not associated with *C. trachomatis*, *M. genitalium*, or *N. gonorrhoeae*, there was weak evidence of an association of presence of *T. vaginalis* with FGS, which remained after adjusting for age. This association was also present when participants with FGS and probable FGS were combined and was strengthened in the ad hoc analyses of participants with higher-burden FGS.

We performed 2 ad hoc analyses. High-intensity *S. haematobium* infection, in the absence of evaluation for FGS, has been associated with higher cervicovaginal alpha diversity [11]. Thus, first we investigated whether *Schistosoma* DNA concentrations might be associated with the cervicovaginal microbiota in 16 participants with a higher FGS burden, indicated by moderate/high genital *Schistosoma* DNA concentrations. In this ad hoc analysis, we found that the *G. vaginalis* log-concentration mean was higher in women with a higher FGS burden. Participants underwent CVL when they were not menstruating, and we have previously described that 66.2% (139/210) of women in this cohort had detectable CVL hemoglobin. Iron sources, like hemoglobin, are often required for bacterial growth [33]. *G. vaginalis* is well adapted to harvest iron from the environment [34], and higher concentrations of *G. vaginalis* coincide with menses [33]. Cervical tissue in women with FGS is more vascularized than non-egg-containing tissue, and thus the abnormal cervical vessels and contact bleeding seen in clinical FGS provide a plausible link to increased concentrations of *G. vaginalis* in high-burden FGS [35].

In a second ad hoc analysis, we examined participants with multiple qPCR-positive genital specimens as a potential proxy marker of high-burden FGS and found that reduced a *L. iners* concentration (median and log mean) was associated with high-burden FGS. The cervicovaginal microbiota was characterized with 16S rRNA sequencing in Tanzanian women with *S. haematobium* infection (n = 16). Although power was limited, women with high-intensity *S. haematobium* infection had reduced abundance of *L. iners* compared with women with low-intensity infection, albeit without evidence of a difference (*P* = .39) [11]. Further research is needed to evaluate the relationship between the presence and concentration of *L. iners* in FGS.

In both ad hoc analyses, we found that presence of *T. vaginalis* was higher among the participants with a higher FGS burden. Our finding supports results from a small South African study (n = 45) that reported an association between FGS (diagnosed by identification of sandy patches on colposcopy) and presence of *T. vaginalis* in young women (ages 15–23) [36]. Acquisition of *T. vaginalis* and *S. haematobium* may share common risk factors, like age and socioeconomic status [18]. We have previously described higher FGS prevalence among younger age groups [18]. Epidemiologic data from Zambian adolescents, sex workers, and pregnant women (aged 13–45 years) describe a *T. vaginalis* prevalence between 24.6% and 33.2% [37], consistent with the prevalence in our population (24.9%). While *S. haematobium* is geographically restricted to Africa and the Middle East, it is associated with poverty [38] and is acquired through contact with cercariae-infested fresh water [1]. *T. vaginalis* is primarily sexually transmitted, is prevalent worldwide, with the highest prevalence in women from low-income countries [39]. Both *S. haematobium* [40] and *T. vaginalis* infection [41, 42] have been reported as risk factors for HIV-1 acquisition, although these associations are not universally reported [43]. Additionally, *T. vaginalis* may influence HIV-1 transmission. Studies in women not uniformly receiving antiretroviral therapy (ART) with *T. vaginalis* show a decline in genital HIV-1 shedding after metronidazole therapy [44–46]. However, this decline in cervical HIV-1 shedding was not seen in a study of Kenyan women receiving ART [47]. The highest global *T. vaginalis* incidence rates have been reported in Africa [39]. Overlapping synergies between *T. vaginalis* and *S. haematobium* cervicovaginal pathogenesis may begin with, but are not limited to, disruption of the cervicovaginal epithelium [48]. Both parasites have been associated with characteristic cervicovaginal manifestations [17, 49], the “strawberry cervix” in *T. vaginalis* [49] and sandy patches, rubbery papules, and abnormal blood vessels in FGS [17]. Breaches in the cervicovaginal epithelium in *T. vaginalis* infection potentially represent one mechanism of HIV-1 vulnerability in what is likely a multifactorial cascade, including modulating the cervicovaginal immune environment [50–52], disabling innate immunity [51], and disrupting the local microbiota [52]. To disentangle the association between *S. haematobium*, *T. vaginalis*, and HIV-1, future studies using sensitive molecular methods for both parasites are needed. Additionally, macrophage polarization can be influenced by the local immune environment, schistosomes, and *T. vaginalis* [53, 54]. We have previously shown that, compared with FGS-negative women, high-burden FGS is associated with higher concentrations of cervicovaginal Th2 cytokines [32]. A mouse model of urogenital *S. haematobium* infection suggested that the Th2 immune environment may be associated with delayed pathogen clearance [55]. Thus, further research is needed regarding the interaction between the immune environment and macrophage phenotypes in FGS and their role in potentially influencing *T. vaginalis* persistence.

Our study is the first to evaluate the cervicovaginal microbiota and STI in FGS defined by qPCR. This is particularly relevant in a population of sexually active, nonpregnant women in the context of high HIV-1 prevalence. However, there are also some relevant limitations. FGS does not have an accepted diagnostic reference standard, and the sensitivity of cervicovaginal PCR for the detection of *Schistosoma* DNA is imperfect. Thus, studies using *Schistosoma* DNA to detect FGS may be subject to misclassification bias. To maximize sensitivity, we used the results of 3 genital specimens. This decision was supported by previous work showing a sensitivity of 80.0% (61.4%–92.3%) with a specificity of 100.0% (99.3%–100.0%) when vaginal and cervical swab PCR were compared with any positive genital PCR (CVL, cervical swab, or vaginal swab) as the reference standard [18]. The study was conducted in an urban location with relatively low *S. haematobium* prevalence; thus the numbers of FGS cases in the primary and ad hoc analyses were small, limiting precision in the effect sizes. This analysis also included multiple statistical comparisons; thus, we focused on the species that showed a consistent pattern of association across primary and ad hoc analyses, rather than overinterpreting significance testing for any 1 species in isolation. Evidence for these associations in the ad hoc analyses should be viewed as hypothesis-generating. Additionally, the cross-sectional study design limited our ability to assess FGS duration and the long-term impact on the prevalence and concentrations of key species or STIs. There were a number of behavioral and biological factors that were not measured in our study including tobacco use [56], viral STIs (human papillomavirus and herpes simplex virus–2) [56], and intravaginal cleansing practices [57]. As these factors may be associated with the cervicovaginal microbiota, we cannot exclude residual or unmeasured confounding. Genital swabs were self-collected by participants, raising the potential for false-negative genital swabs. In future work, β-globin PCR could be implemented as a positive control to confirm the presence of human DNA [18]. Lastly, we defined FGS by *Schistosoma* DNA detection on qPCR; however, we cannot exclude that cervicovaginal qPCR detected *S. haematobium* eggs from a sexual partner’s semen [18].

In conclusion, we report weak evidence of an association between presence of *T. vaginalis* and FGS, with a consistently stronger association in women with a higher burden FGS infection. Additional research is needed to understand the interactions between *S. haematobium* and *T. vaginalis*, especially regarding HIV-1 vulnerability.

## Supplementary Material

ofab438_suppl_Supplementary_Figure

ofab438_suppl_Supplementary_Tables
