# Supplementary material for: Association of Female Genital Schistosomiasis With the Cervicovaginal Microbiota and Sexually Transmitted Infections in Zambian Women
Source: Open Forum Infect Dis. 2021 Aug 22;8(9):ofab438. doi: 10.1093/ofid/ofab438 (PMC8454507; doi:10.1093/ofid/ofab438)
Supplement: ofab438_suppl_Supplementary_Figure [file ofab438_suppl_supplementary_figure.docx]

**S1 Figure – Causal diagram describing** **the association between FGS and a concentration change of cytokines and chemokines**

**
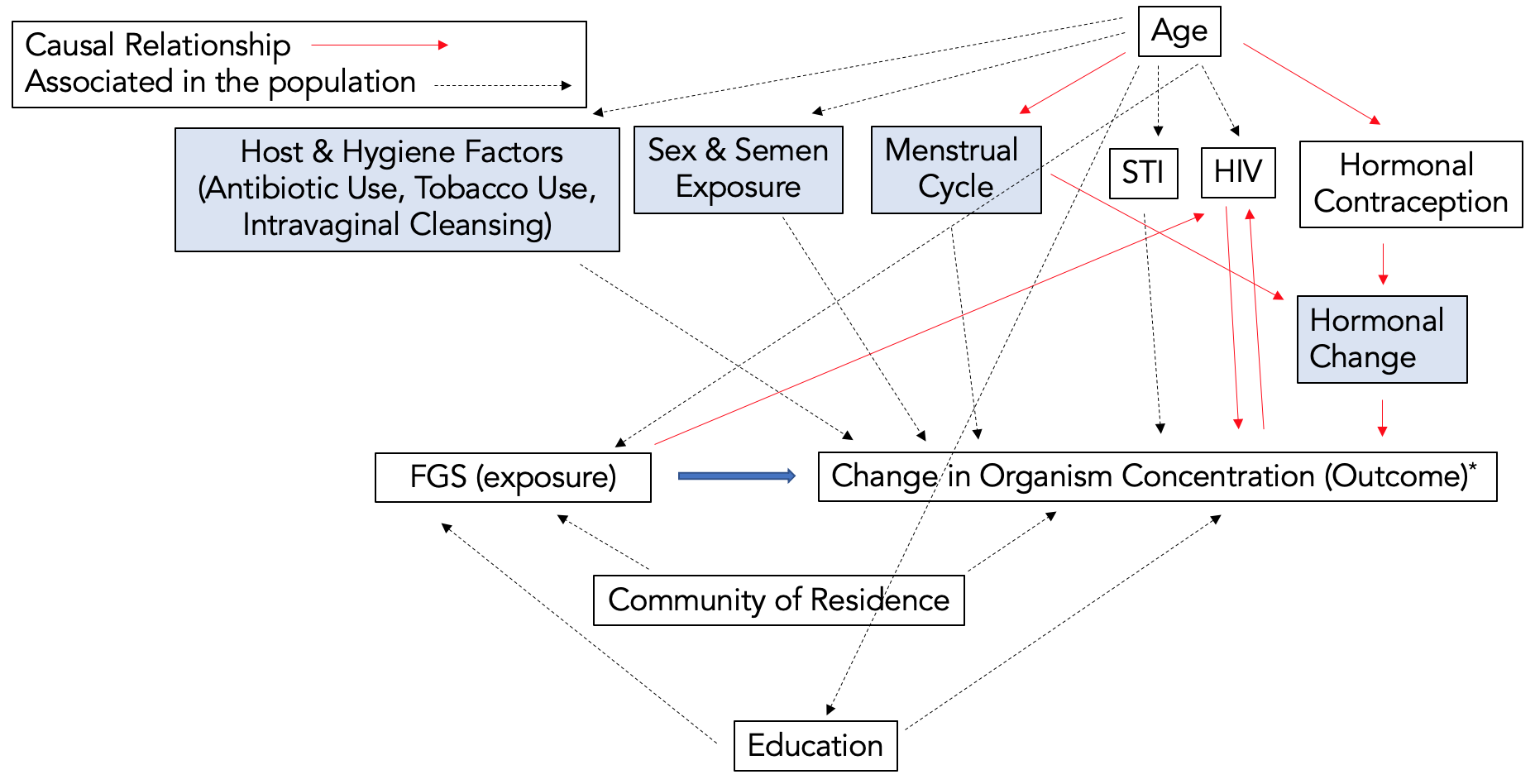
**

**S2-Figure – Study Flow Sheet**


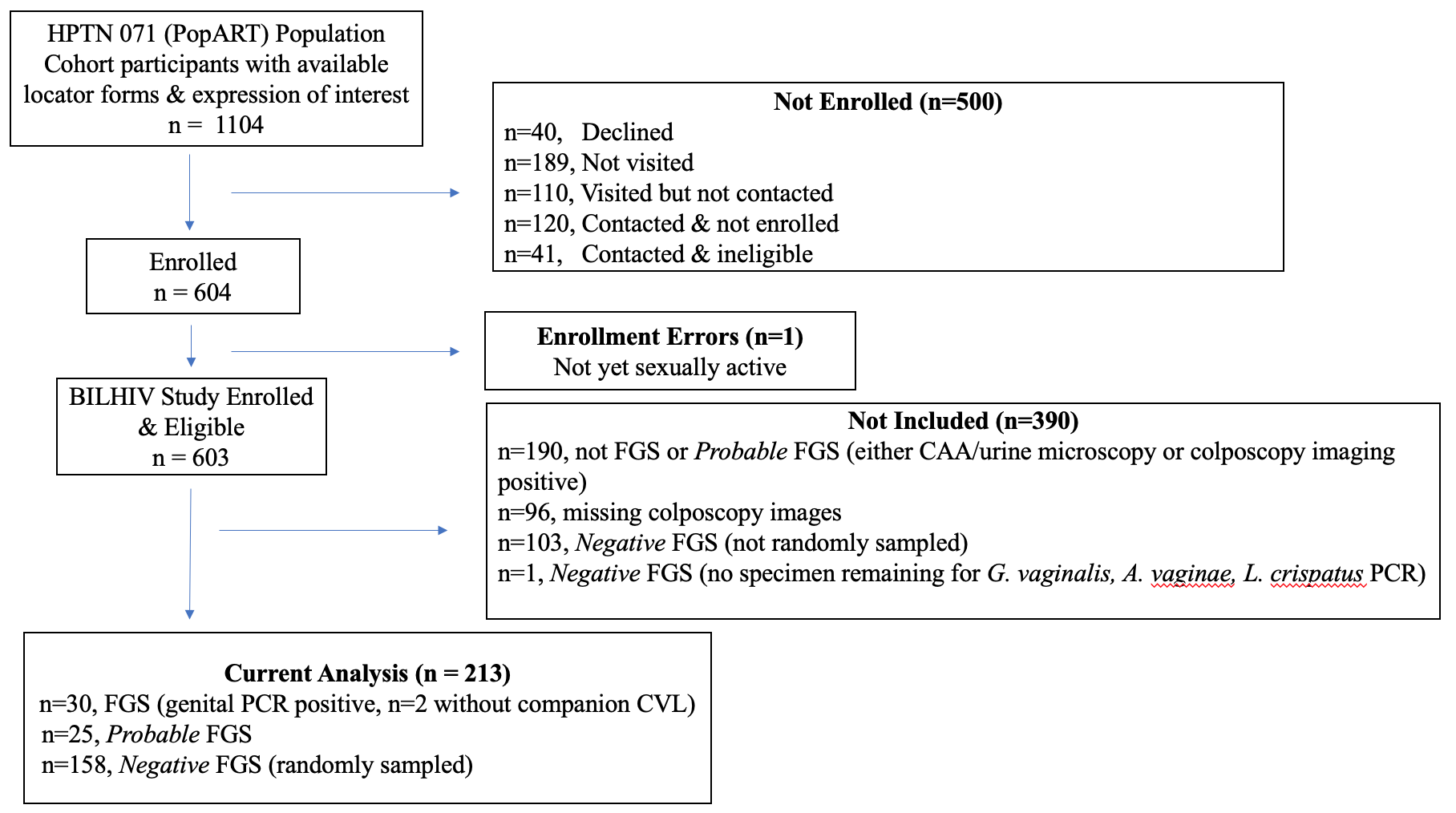


There were 262 women diagnosed as *Negative*FGS of whom 158 were randomly sampled for comparison with the FGS and *Probable* FGS groups
