# Supplementary material for: Association of Female Genital Schistosomiasis With the Cervicovaginal Microbiota and Sexually Transmitted Infections in Zambian Women
Source: Open Forum Infect Dis. 2021 Aug 22;8(9):ofab438. doi: 10.1093/ofid/ofab438 (PMC8454507; doi:10.1093/ofid/ofab438)
Supplement: ofab438_suppl_Supplementary_Tables [file ofab438_suppl_supplementary_tables.docx]

**S1 Table – Crude and adjusted associations of** presence and mean log-transformed concentrations of vaginal *Lactobacilli*, other cervicovaginal microbiota, and sexually transmitted infection with female genital schistosomiasis status

| **Organism** | **n(%) and Concentration^*^** | **All Participants (n=213)** | **FGS Negative**  **(n=158)** | **FGS**  **(n=30)** | **Crude Regression Estimate^**^ (OR, difference in means)** | **p-value**^††^ | **Adjusted Regression Estimate**^†††^ **(OR, difference in means)** | **LRT**  **p-value** |
| --- | --- | --- | --- | --- | --- | --- | --- | --- |
|  |  |  |  |  |  |  |  |  |
| *L. crispatus* | Presence**^**^** | 69 (32.4) | 49 (31.0) | 13 (43.3) | 1.70 (0.77 – 3.77) | 0.19 | 1.68 (0.74 – 3.79) | 0.22 |
|  | Log conc mean ^†^ | 16.01 | 16.38 | 14.82 | -1.56 (-3.90 – 0.78) | 0.19 | -1.93 (-4.57 – 0.71) | 0.13 |
| *L. iners* | Presence**^**^** | 156 (73.2) | 113 (71.5) | 24 (80.0) | 1.60 (0.61 – 4.16) | 0.34 | 1.68 (0.74 – 3.79) | 0.22 |
|  | Log conc mean ^†^ | 18.93 | 18.98 | 18.36 | -0.62 (-1.88 – 0.64) | 0.33 | -0.53 (-1.83 – 0.77) | 0.41 |
| *G. vaginalis* | Presence**^**^** | 156 (73.2) | 115 (72.8) | 23 (76.7) | 1.23 (0.49 – 3.07) | 0.66 | 1.22 (0.49 – 3.05) | 0.67 |
|  | Log conc mean ^†^ | 15.85 | 15.74 | 15.91 | -0.17 (-1.08 – 1.41) | 0.79 | 0.24 (-1.05 – 1.53) | 0.71 |
| *A. vaginae* | Presence**^**^** | 152 (71.4) | 112 (70.9) | 19 (63.3) | 0.71 (0.31 – 1.61) | 0.41 | 0.71 (0.31 – 1.61) | 0.42 |
|  | Log conc mean ^†^ | 17.31 | 17.33 | 17.06 | -0.27 (-1.51 – 0.96) | 0.66 | -0.18 (-1.44 – 1.07) | 0.77 |
| *T. vaginalis* | Presence**^**^** | 53 (24.9) | 34 (21.5) | 11 (36.7) | 2.11 (0.92 – 4.86) | 0.08 | 2.09 (0.90 – 4.83) | 0.09 |
|  | Log conc mean ^†^ | 10.39 | 10.88 | 10.19 | -0.69 (-4.16 – 2.77) | 0.69 | -0.65 (-4.47 – 3.17) | 0.71 |
| ***Vaginal microbiota with prevalence <20%*** | | |  |  |  |  |  |  |
| *N. gonorrheae* | Presence | 13 (6.1) | 12 (7.6) | 0.0 (0) | Not applicable | 0.22 | Not applicable | NA |
| *C. trachomatis* | Presence**^**^** | 17 (8.0) | 13 (8.2) | 3 (10.0) | 1.24 (0.33 – 4.64) | 0.72 | 1.20 (0.32 – 4.54) | 0.79 |
| *M. genitalium* | Presence | 8 (3.8) | 7 (4.4) | 0 (0.0) | Not applicable | 0.60 | Not applicable | NA |
| *C. albicans* | Presence**^**^** | 12 (5.63) | 8 (5.1) | 2 (6.7) | 1.34 (0.27 – 6.64) | 0.66 | 1.33 (0.27 – 6.61) | 0.73 |

^*^concentrations are expressed in genome equivalents/mL

^**^ for presence/absence, regression estimates are odds ratios (OR) generated by logistic regression, for log concentration mean, regression estimates are differences in means generated by linear regression

^††^ For species with >20% prevalence, p-values for presence and log concentration mean are from chi-squared test and t-test, respectively. For species with <20% prevalence, p-values for presence are from Fisher’s exact test.

^†††^presence/absence logistic regression adjusted for age, log concentration mean linear regression adjusted for age, community of residence and education

**S2 Table –** Presence and mean log-transformed concentrations of vaginal *Lactobacilli*, other cervicovaginal microbiota, and sexually transmitted infection with crude and adjusted associations comparing participants with FGS or probable FGS (combined) with those who were FGS negative

| **Organism** | **n(%) and Concentration^*^** | **All Participants**  **(n=213)** | **FGS Negative**  **(n=158)** | **FGS & Probable FGS**  **(n=55)** | **p-value**^**^ |
| --- | --- | --- | --- | --- | --- |
|  |  |  |  |  |  |
| *L. crispatus* | Presence | 69 (32.4) | 49 (31.0) | 20 (36.4) | 0.47 |
|  | Median (IQR) | 8.7x10^6^ (2.6x10^5^ – 4.2x10^8^) | 1.1x10^7^ (2.6x10^5^ – 7.0x10^8^) | 1.3x10^6^ (2.3x10^5^ – 2.3x10^7^) | 0.24 |
|  | Log concentration mean | 16.01 | 16.38 | 15.11 | 0.21 |
| *L. iners* | Presence | 156 (73.2) | 113 (71.5) | 43 (78.2) | 0.34 |
|  | Median (IQR) | 2.7x10^8^ (3.8x10^7^ – 1.5x10^9^) | 2.8x10^8^ (3.9x10^7^ – 1.6x10^9^) | 2.7x10^8^ (3.3x10^7^ – 1.4x10^9^) | 0.73 |
|  | Log concentration mean | 18.93 | 18.98 | 18.81 | 0.74 |
| *G. vaginalis* | Presence | 156 (73.2) | 115 (72.8) | 41 (74.6) | 0.80 |
|  | Median (IQR) | 7.7x10^6^ (8.3x10^5^ – 5.2x10^7^) | 8.1x10^6^ (7.7x10^5^ – 4.8x10^7^) | 5.9x10^6^ (1.1x10^6^ – 1.0x10^8^) | 0.52 |
|  | Log concentration mean | 15.85 | 15.74 | 16.14 | 0.42 |
| *A. vaginae* | Presence | 152 (71.4) | 112 (70.9) | 40 (72.7) | 0.79 |
|  | Median (IQR) | 5.8x10^7^ (8.2x10^6^ – 2.1x10^8^) | 5.8x10^7^ (8.7x10^6^ – 2.0x10^8^) | 6.1x10^7^ (4.7x10^6^ – 2.7x10^8^) | 0.97 |
|  | Log concentration mean | 17.31 | 17.33 | 17.25 | 0.87 |
| *T. vaginalis* | Presence | 53 (24.9) | 34 (21.5) | 19 (34.6) | 0.05 |
|  | Median (IQR) | 4.2x10^4^ (173.0 – 2.3x10^6^) | 1.7x10^5^ (56.9 – 6.3x10^6^) | 6.0x10^3^ (361.0 – 4.5x10^5^) | 0.35 |
|  | Log concentration mean | 10.39 | 10.88 | 9.52 | 0.35 |
| ***Vaginal microbiota with prevalence <20%*** | | |  |  |  |
| *N. gonorrheae* | Presence | 13 (6.1) | 12 (7.6) | 1 (1.8) | 0.19 |
| *C. trachomatis* | Presence | 17 (8.0) | 13 (8.2) | 4 (7.3) | 1.0 |
| *M. genitalium* | Presence | 8 (3.8) | 7 (4.4) | 1 (1.8) | 0.68 |
| *C. albicans* | Presence | 12 (5.63) | 8 (5.1) | 4 (7.3) | 0.51 |

^*^concentrations are expressed in genome equivalents/mL

^**^ For species with >20% prevalence, p-values for presence, median (IQR) and log concentration mean from chi-squared test, ranksum test and t-test, respectively. For species with <20% prevalence, p-values for presence are from Fisher’s exact test.

**Further details of home and clinic-based sample collection**

*This section provides additional detail to the details of home and clinic-based sample collection provided in the main manuscript.*

The BILHIV study home visit included written informed consent, a questionnaire, genital self-sampling (cervical and vaginal), and collection of a urine specimen, as previously described [1]. Specimens were placed in cool boxes for transportation and were stored in the laboratory at -80°C. Vaginal and cervical swab specimens were used for PCR detection of *Schistosoma* species; cervical swabs were used for characterization of the microbiota and STI by quantitative PCR (qPCR); urine was used for detection of circulating anodic antigen (CAA) and microscopic evaluation of the urine for *S. haematobium* eggs.

Enrolled women who were not currently menstruating were invited to attend Livingstone Central Hospital cervical cancer screening clinic, where one of two trained midwives performed cervicovaginal lavage (CVL), which was used for *Schistosoma* PCR. After speculum insertion, a bulb syringe was used to flush normal saline (10 mL) continuously across the cervix and vaginal walls for one minute. CVL fluid was collected from the posterior fornices and stored temporarily in a refrigerator (4 °C) on ice until transfer to the laboratory, where specimens were stored at -80°C. All specimens were shipped on dry ice to Leiden University Medical Center (LUMC).

Cervicovaginal images were captured with a portable colposcope (MobileODT, Tel Aviv, Israel) and were evaluated by one author (EFK) for the presence of any of the four recognized FGS cervicovaginal manifestations: grainy sandy patches, homogenous yellow sandy patches, rubbery papules, and abnormal blood vessels [2]. Women with at least one of these manifestations [2] and women with any positive urine or genital *Schistosoma* diagnostic were treated free of charge with 40 mg/kg praziquantel. Testing for STI was not performed at the point-of-care and participants with suspected STI were offered syndromic management, as per local guidelines [3].

**Urine microscopy and Circulating Anodic Antigen**

After aliquoting for CAA quantification, urine was centrifuged in 15 mL aliquots and examined by microscopy for *S. haematobium* eggs within 24 hours. The participant was considered positive if a pellet contained at least one *S. haematobium* egg, as previously described [1]. A lateral flow assay utilizing up-converting reporter particles for the quantification of CAA was performed on urine samples, as previously described [1, 4]. CAA levels reflect the burden of live schistosomes and decline after successful treatment with praziquantel [5, 6]. Analyzing the equivalent of 417 μL urine (wet reagent, UCAA***hT***417), a test result indicating a CAA value of >0.6 pg/mL was considered positive [6].

**PCR for detection of *Schistosoma* DNA**

DNA extraction and detection of the *Schistosoma*-specific internal-transcribed-spacer-2 (ITS2) target by real-time PCR were performed at LUMC, using a custom automated liquid handling station (Hamilton, Switzerland), as previously described [1, 7]. DNA extraction of 200 μL of CVL, cervical or vaginal swab fluid was done with QIAamp spin columns (QIAGEN, Benelux; Venlo, The Netherlands) according to the manufacturer’s guidelines. Schistosome DNA amplification and detection were performed with the CFX96 Real Time PCR Detection System and BioRad CFX software (BioRad, California, USA). DNA extracted from cervical swabs was transported to Ghent University for further analysis.

**Cervicovaginal microbiota characterization and STI detection**

DNA from cervical swabs were used to quantified key markers of vaginal health (*Lactobacillus crispatus*), markers of a non-optimal cervicovaginal microbiota (*Gardnerella vaginalis* and *Atopobium* *vaginae*), *Lactobacillus iners* (a highly prevalent lactobacillus with an enigmatic role), *Candida spp* and STI (*Chlamydia trachomatis*, *Neisseria gonorrhoeae*, *Mycoplasma genitalium*, and *Trichomonas vaginalis)* by means of qPCR at the Laboratory Bacteriology Research (Ghent University, Ghent, Belgium).

For the *L. crispatus*, *L. iners*, *G. vaginalis*, *A. vaginae* and *Candida species* qPCR, a total reaction volume of 10 µL was prepared by the addition of 2 µL of DNA extract of the CVL, positive control (i.e., DNA of the corresponding type strain (listed for each species in Table 1)) or negative control (HPLC water) to 8 µL of reaction mixture. The final reaction mixture of each qPCR consisted of primers (listed in Table 2) in 1X LightCycler 480 SYBR Green I master mix in HPLC water. Reaction conditions for *A. vaginae* were pre-incubation for 10 min at 95 °C, followed by 40 cycles of 15 s at 95 °C, 20 s at 62 °C and 40 s at 72 °C, for *G. vaginalis* pre-incubation 5 min at 95 °C, followed by 40 cycles of 15 s at 95 °C, 30 s at 56 °C and 30 s at 72 °C, for *L. crispatus* pre-incubation for 10 min at 95 °C, followed by 40 cycles of 15 s at 95 °C, 30 s at 60 °C and 30 s at 72 °C, for *L. iners* pre-incubation for 10 min at 95 °C, followed by 40 cycles of 10 s at 95 °C, 20 s at 50 °C and 4 s at 72 °C and *Candida* species by pre-incubation for 10 min at 95 °C, followed by 45 cycles of 20 s at 95 °C, 30 s at 55 °C and 30 s at 72 °C. High resolution melting curves were generated for each species by melting all amplified double stranded DNA at 95 °C for 5 s, followed by renaturating DNA for 30 s at 50 °C (*A. vaginae*), 60 s at 55 °C (*G. vaginalis*), 60 s at 60 °C (*L. crispatus*, *L. iners* and *Candida spp.*), whereafter the temperature was increased to 97 °C at a ramp rate of 0.02 °C per s. *Candida* speciation was determined based on the melting peak temperatures as previously described [8].

We quantified *C.* *trachomatis*, *N. gonorrhoeae*, *M. genitalium*, and *T. vaginalis* using the S-DiaCTNG^TM^ (for *C. trachomatis* and *N. gonorrhea*) and S-DiaMGTV^TM^ (for *M. genitalium* and *T. vaginalis*) (both Diagenode Diagnostics, Seraing, Belgium) according to the manufacturer’s instructions.

To quantify each of the target species, standard curves were constructed from a tenfold dilution series of DNA from *C. trachomatis* (ATCC VR-571B), *N. gonorrhoeae*, *M. genitalium* (ATCC G37), and *T. vaginalis* (ATCC 50148) (all commercially purchased). Genomic DNA from *N. gonorrhoeae* (ATCC 43069), *A. vaginae*, *G. vaginalis*, *L. crispatus*, *L. iners* and *C. albicans* was obtained after culturing the strains according to conditions from Table 1 and extracting DNA from colonies using Roche High Pure DNA Purification kit (Roche). All DNA concentrations were determined using NanoDrop (Thermo Fisher scientific, Erembodegem, Belgium). The genomic concentrations were calculated using the described genomic sizes of the type strains. Both the standard curves and samples were run in duplicate. The bacterial, fungal and protozoan concentrations were expressed as genome equivalents per mL (ge/mL) [9].

All non-schistosome qPCR assays were performed using the LightCycler480® and the LightCyclerR 480 Software Version 1.5 (Roche, Basel, Switzerland).

Table 1: Strains used in this study

| Species | Strain | Culture conditions |
| --- | --- | --- |
| *Atopobium vaginae* | CCUG 38953^T^ | Anaerobe, 37 °C, TSA plates |
| *Gardnerella vaginalis* | LMG 7832^T^ | Anaerobe, 37 °C, chocolate agar plates |
| *Lactobacillus crispatus* | LMG 9479^T^ | Anaerobe, 37 °C, NYC+ HS agar plates |
| *Lactobacillus iners* | ACS-049-V-Sch2 | Anaerobe, 37 °C, NYC+ HS agar plates |
| *Candida albicans* | ATCC 90028 | Aerobe, 30 °C, CHROMID® Candida agar |
| *Chlamydia trachomatis* | ATCC VR-571B | N/A |
| *Neisseria gonorrhoeae* | ATCC 43069 | 35°C ±1°C for 5 days on chocolate agar (Becton Dickinson) |
| *Mycoplasma genitalium* | ATCC G37 | N/A |
| *Trichomonas vaginalis* | ATCC 50148 | N/A |

Table 2: Primers used in this study

| Species | Forward primer (5’-3’) | Final concentration (µM) | Reverse Primer (5’-3’) | Final concentration (µM) |
| --- | --- | --- | --- | --- |
| *Atopobium vaginae* | CCCTATCCGCTCCTGATACC | 0.7 | CCAAATATCTGCGCATTTCA | 0.7 |
| *Gardnerella vaginalis* | TATTATAACTAAAGCTGCTG | 0.5 | CGCCACTATAGTCG | 0.5 |
| *Lactobacillus crispatus* | AGCGAGCGGAACTAACAGATTTAC | 0.1 | AGCTGATCATGCGATCTGCTT | 0.1 |
| *Lactobacillus iners* | GTCTGCCTTGAAGATCGG | 0.2 | ACAGTTGATAGGCATCATC | 0.2 |
| *Candida* spp. | GTGAATCATCGAATCTTTGAAC | 0.5 | TCCTCCGCTTATTGATATGC | 0.5 |

**REFERENCES**

1. Sturt AS, Webb EL, Phiri CR, et al. Genital self-sampling compared with cervicovaginal lavage for the diagnosis of female genital schistosomiasis in Zambian women: The BILHIV study. PLoS Negl Trop Dis **2020**; 14:e0008337.

2. World Health Organization. Female genital schistosomiasis: a pocket atlas for clinical health-care professionals. World Health Organization. 2015. Available at, <http://www.who.int/iris/handle/10665/180863> [accessed January 12, 2021].

3. Zambian Ministry of Health. Guidelines for the Etiological and Clinical Management of Sexually Transmitted Infections in Zambia, 2017.

4. Corstjens PL, De Dood CJ, Kornelis D, et al. Tools for diagnosis, monitoring and screening of Schistosoma infections utilizing lateral-flow based assays and upconverting phosphor labels. Parasitology **2014**; 141:1841-55.

5. van Lieshout L, Polderman AM, Deelder AM. Immunodiagnosis of schistosomiasis by determination of the circulating antigens CAA and CCA, in particular in individuals with recent or light infections. Acta Trop **2000**; 77:69-80.

6. Corstjens P, de Dood CJ, Knopp S, et al. Circulating Anodic Antigen (CAA): A Highly Sensitive Diagnostic Biomarker to Detect Active Schistosoma Infections-Improvement and Use during SCORE. Am J Trop Med Hyg **2020**; 103:50-7 doi:10.4269/ajtmh.19-0819.

7. Obeng BB, Aryeetey YA, de Dood CJ, et al. Application of a circulating-cathodic-antigen (CCA) strip test and real-time PCR, in comparison with microscopy, for the detection of Schistosoma haematobium in urine samples from Ghana. Annals of tropical medicine and parasitology **2008**; 102:625-33.

8. Duyvejonck H, Cools P, Decruyenaere J, et al. Validation of High Resolution Melting Analysis (HRM) of the Amplified ITS2 Region for the Detection and Identification of Yeasts from Clinical Samples: Comparison with Culture and MALDI-TOF Based Identification. PloS one **2015**; 10:e0132149.

9. Jespers V, van de Wijgert J, Cools P, et al. The significance of Lactobacillus crispatus and L. vaginalis for vaginal health and the negative effect of recent sex: a cross-sectional descriptive study across groups of African women. BMC Infect Dis **2015**; 15:115.
